# Reported analgesic administration to rabbits undergoing experimental surgical procedures

**DOI:** 10.1186/1746-6148-7-12

**Published:** 2011-02-21

**Authors:** Claire A Coulter, Paul A Flecknell, Matthew C Leach, Claire A Richardson

**Affiliations:** 1 Comparative Biology Centre, Newcastle University, Newcastle upon Tyne, UK

## Abstract

**Background:**

It has become widely accepted that whenever animals are used in scientific procedures, the 3Rs principle of replacement, reduction and refinement described by William Russell and Rex Burch should be adhered to. Animals should be replaced with non-sentient alternatives if possible, the number of animals used should be reduced and experimental procedures should be refined to minimise pain, suffering and distress. Administration of analgesic agents to animals undergoing surgical procedures is a refinement used to alleviate pain. In this study, a structured literature review was carried out to examine current trends in analgesic administration to rabbits undergoing experimental surgical procedures.

**Results:**

128 papers from 51 peer-reviewed journals were selected for inclusion in this review. Reporting administration of systemic analgesia to rabbits in peer-reviewed scientific papers increased significantly from 16% to 50% between 1995-1997 and 2005-2007 (*P *< 0.001). Papers that reported ethical approval were more likely than papers that did not specify approval to report systemic analgesic administration (*P *< 0.001). When systemic analgesics were administered, buprenorphine was the most frequently used agent and non-steroidal anti-inflammatory drugs were used less frequently than opioids in both time periods.

**Conclusions:**

Although this review provides evidence that systemic analgesic administration to rabbits undergoing surgical procedures is increasing, rabbits do not always receive analgesia when they undergo experimental surgery. Other practices in rabbit perioperative care that could be improved, highlighted by this survey include: 1) changing the timing of analgesic administration by giving systemic analgesics pre- or perioperatively rather than only postoperatively, 2) using multimodal analgesia when pain is likely to be moderate to severe and 3) increasing the use of non-steroidal anti-inflammatory drugs and use of other techniques such as epidural analgesia particularly for orthopaedic procedures.

## Background

The 3Rs principle of replacement, reduction and refinement has become widely accepted, and is incorporated into the legislation governing the use of animals in several EU member states and throughout the world. This study was undertaken to gain an indication as to how widely one clear refinement, administration of analgesics to prevent pain in animals undergoing surgery, had been implemented in rabbits.

When used in biomedical research, rabbits are most frequently used in toxicity studies [1], for antibody production [2] and as surgical models, particularly in the field of orthopaedics [3]. Amongst the countries that annually report the number of animals used in scientific procedures, there is a general downward trend in the numbers of rabbits used [4-6]. For example, in Canada, 18 155 laboratory rabbits (1.2% of all animals used in scientific procedures) were used in 1997 compared to 8 838 (0.4% of animals used) in 2007 [7].

The overall aim of this study was to follow up on a smaller previous study [8] and examine whether reported practices in analgesic administration to laboratory rabbits undergoing experimental surgery are optimal. Specifically we aimed to: 1) examine whether systemic analgesic administration to rabbits is increasing, 2) evaluate changes in the administration of analgesics in various forms including local anaesthesia and anaesthetic agents with analgesic components and 3) examine trends in the reporting of other animal details such as reporting ethical approval and number/gender of rabbits used.

## Results

128 papers, 64 from each time period (1995-1997 and 2005-2007) were selected from 51 journals for inclusion in this review (Additional file 1). 25 papers from 1995/2005, 24 papers from 1996/2006 and 15 papers from 1997/2007 were included.

In both time periods, the surgical procedures most common in laboratory rabbits were orthopaedic surgeries followed by skin incisions. Orthopaedic procedures were described in 50% of papers from 1995 to 1997 and 57% of papers from 2005 to 2007. Skin incisions were described in 25% of papers from both times periods.

Administration of systemic analgesia to rabbits undergoing experimental surgical procedures increased significantly (*P *< 0.001) from 16% to 50% between the two time periods. Buprenorphine was the most commonly reported systemic analgesic administered in both 1995-1997 (60%) and in 2005-2007 (70%) (Table 1). The reported dose range of buprenorphine varied from 0.01 mg/kg to 0.2 mg/kg (Table 1). The overall use of non-steroidal anti-inflammatory drugs (NSAIDs) was similar in the two time periods (20% in 1995-1997, 21% in 2005-2007).

**Table 1:** Reporting of systemic analgesic administration classified by agent.

Class of analgesic	Analgesic	1995-1997	2005-2007
**Opioid**	Buprenorphine	6 (60%) *4* *[0.04-0.1 mg/kg]*	23 (70%) *13* *[0.01-0.2 mg/kg]*

	Butorphanol	2 (20%) *1* *[0.2 mg/kg]*	2 (6%) *1* *[0.04 mg/kg]*

	Fentanyl	0	1 (3%) *0*

**NSAIDs**	Carprofen	0	4 (12%) *3 [4-5 mg/kg]*

	Flunixin	1 (10%) *0*	0

	Metamizol	0	2 (6%) *0*

	Paracetamol	1 (10%) *0*	1 (3%) *0*

**Total**		**10** ***5***	**33**

Number (proportion) of papers reporting specific systemic analgesic. Number of papers reporting dose of analgesic in italics *[dose range]*.

NSAID administration for orthopaedic procedures (the most common experimental surgical procedure in both time periods) was reported in one paper which from 1995-1997 where flunixin was administered (e.g. 3% of rabbits undergoing orthopaedic procedures receiving a NSAID). From 2005-2007, 5 (13.5%) of the papers describing orthopaedic procedures reported NSAID administration: carprofen (n = 3), metamizol (n = 1) and paracetamol (n = 1).

When procedures were classified according to their potential to be painful, the number of procedures in each category did not differ significantly between 1995-1997 and 2005-2007. In both time periods, rabbits that underwent the most potentially painful procedures were not more likely to receive systemic analgesia than rabbits that underwent less potentially painful procedures. For example, in 1995-1995 22% of papers describing the least painful procedures (craniotomies and skin incisions) compared to 13% of papers describing the most painful procedures (thoracotomies and orthopaedic procedures) reported systemic analgesic administration. Similarly in 2005-2007, 44% of papers describing the least painful procedures compared to 51% describing the most painful procedures reported systemic analgesic administration.

When systemic analgesics were reported, all papers from both time periods specified the timing of administration. In 1995-1997 20% of systemic analgesics were administered perioperatively compared to 80% postoperatively. In 2005-2007 9% of systemic analgesics were administered preoperatively, 9% perioperatively, 66% postoperatively, 6% both preoperatively and postoperatively and 9% both perioperatively and postoperatively.

The proportion of rabbits that were reported to have received some form of analgesia (either a systemic analgesic, an anaesthetic regimen with an analgesic component and/or a local anaesthetic) was similar in the two time periods (83% in 1995-1997 and 87% in 2005-2007). The use of 'agents with analgesic properties' are summarised in Table 2. Anaesthetic regimens with an analgesic component were more commonly reported in papers published between 2005 and 2007 (88%) compared to 1995 and 1997 (70%) (*P *= 0.015) and were commonly combined with the use of a systemic analgesic, whereas use of local anaesthetic agents decreased significantly between time periods (38% to 6%) (*P *= 0.001) (Table 2).

**Table 2:** Classification of papers by reported use of all analgesic agents.

Analgesic regimen	Number (proportion) of papers specifying this analgesic regimen in review	
	**1995-1997**	**2005-2007**

Systemic analgesia only	0	0

Local anaesthesia only	8 (13%)	0

Anaesthetic with analgesic property only	28 (44%)	21 (33%)

Systemic analgesia and local anaesthesia	0	0

Systemic analgesia and anaesthetic with analgesic properties	7 (11%)	31 (48%)

Local anaesthesia and anaesthetic with analgesic properties	7 (11%)	3 (4%)

Systemic analgesia, local anaesthesia and anaesthetic with analgesic properties	3 (4%)	1 (2%)

No analgesia	11 (17%)	8 (13%)

**Total**	**64**	**64**

None of the papers reported the administration of an opioid analgesic in combination with a non-steroidal anti-inflammatory drug and only one paper published between 2005 and 2007 reported the use of two systemic analgesic agents: buprenorphine and fentanyl. When systemic analgesic agents were used, they were always combined with an anaesthetic regimen with analgesic properties in both time periods (Table 2). Between 1995 and 1997, local anaesthetic agents were frequently the only agents with analgesic properties used. In 2005 to 2007 local anaesthetics were less frequently used and when used usually combined with other agents with analgesic properties (Table 2).

There was a significant increase in the number of papers reporting ethical approval between time periods (*P *< 0.001). 44% of papers from 1995 to 1997 compared to 95% of papers from 2005 to 2007 specified that ethical approval was obtained. Overall, papers that reported ethical approval were also more likely to report the administration of systemic analgesic agents (*P *< 0.001).

The majority of the papers (96%) reported the number of animals used. In both time periods the median number of animals used was similar (1995-1997: median = 22, range = (4, 200); 2005-2007: median = 23, range = (6, 64). Male rabbits were more commonly used than females. From 1995-1997 the gender reported was 34% (males), 22% (females), 10% (both male and females) and 34% (unspecified). From 2005-2007 the gender reported was 43% (males), 18% (females), 4% (males and females) and 35% (unspecified). Neither the gender of rabbits nor the number of animals used differed significantly between time periods.

## Discussion

The reporting of administration of systemic analgesic drugs to laboratory rabbits undergoing surgical procedures is increasing. Unfortunately, even in papers published between 2005 and 2007, not all rabbits undergoing potentially painful surgical procedures received a systemic analgesic agent.

Guidelines on refining experimental surgical procedures involving rabbits summarized from several sources are presented in Table 3. This survey highlights several areas in rabbit care where what is reported in peer-reviewed papers differs from recommended best practice. Factors in the care of rabbits undergoing experimental surgical procedures that will be discussed include: (1) choice of analgesic, (2) dose of analgesic, (3) matching analgesic administration to the severity of the procedure, (4) timing of analgesic, (5) consideration of various pharmacological forms of analgesia, (6) multimodal analgesia and (7) general reporting of experiments involving rabbits.

**Table 3:** Guidelines to refine experimental surgical procedures involving rabbits modified from Stokes *et al*. 2009 [9].

Recommendation	Reference (s)
(1) **Analgesic administration**	

Administer at least one dose of systemic analgesia to all rabbits undergoing recovery surgical procedures that are likely to be painful	Dobromylskj *et al*. [20], Kohn *et al*. [18]

Consider the use of multimodal analgesia when pain is likely to be moderate to severe	Committee on Recognition and Alleviation of Pain in Laboratory Animals [14], Dobromylskj *et al*. [20], Flecknell [13], Kohn *et al*. [18]
	
Consider the use of preemptive analgesia	
	
Match analgesic administration (dose rate, dose intervals and duration of administration) to the severity of the procedure	

(2) **Reporting of experimental procedures involving rabbits**	

Authors should include more information on analgesia and pain assessment in methods of peer-reviewed publications	Hawkins [21]

In editorial policies/'Instructions to authors' editors should request that analgesic administration be specified and if analgesics were withheld to explain why	Hawkins [21], Richardson and Flecknell [22]

Report animal details in sufficient detail	Smith *et al*.[16]

(3) **Future research**	

Future research into effective pain assessment in rabbits	Leach *et al*.[23]

When systemic analgesic administration was reported the choice of the agent administered was generally appropriate for rabbits (Tables 1 and 4). Opioids were reported more frequently than NSAIDs, particularly for orthopaedic procedures. Rabbits are frequently used as models for orthopaedic conditions [3] and 55% of the peer-reviewed papers in this study were orthopaedic studies (compared to only 15% in rodents based on an earlier study [9]). NSAID administration was infrequently reported in the orthopaedic papers included in this survey. Historically there has been a reluctance to administer non-steroidal anti-inflammatory drugs (NSAIDs) due to the effects of NSAIDs on bone healing [10]. Although NSAIDs were often not used in orthopaedic studies they can have very strong analgesic properties particularly in combination with opioid agents and their effects on bone healing are likely to occur only if administered in high doses for relatively long periods [11].

**Table 4:** Suggested analgesic dose ranges for rabbits.

Class of analgesic	Analgesic	Dose range	Reference(s)
**Opioid**	Buprenorphine	0.01-0.05 mg/kg sc or iv, 6-12 hourly	Hawkins [24], Johnston [12], Kohn *et al*. [18]

	Butorphanol	0.1-0.5 mg/kg iv, 4 hourly	Flecknell [13], Kohn *et al*. [18]

**NSAIDs**	Carprofen	4 mg/kg sc or 1.5 mg/kg po	Flecknell [13], Kohn *et al*. [18]

	Meloxicam	0.6-1 mg/kg sc or po	Flecknell [13]

Dose ranges are likely to be subject to revision and are typically based on clinical impression. Ideally dose rates should be based on objective assessment of postoperative pain. Whenever possible, a pain scoring system should be used, so that dose rates can be adjusted according to the animal's response.

sc = subcutaneous injection, iv = intravenous injection, po = by mouth.

When doses of systemic analgesics were reported, they were generally within the recommended range (Tables 1 and 4). One exception was several papers that reported use of buprenorphine at doses considerably higher than those currently recommended (13). Although pain-induced ileus may often be more of a concern than opioid-induced ileus [12], potential adverse effects of analgesics are more likely to occur when recommended dose ranges are exceeded [13].

There was no evidence that rabbits that underwent the potentially most painful procedures were more likely to systemic analgesia than rabbits that underwent less potentially painful procedures in either time period. This is a significant welfare concern as analgesic administration should be matched to the severity of the experimental procedure. Although there was no relationship between probability of receiving a systemic analgesic and the severity of the experimental procedures, there was a relationship between reporting systemic analgesic administration and in reporting ethical review permissions. Papers that reported ethical approval were more likely to report systemic analgesic administration. This may represent a positive influence of local ethical review or differences in animal care regulation.

The administration of systemic analgesic agents was rarely reported to occur pre- or perioperatively and systemic analgesics were usually administered postoperatively in both time periods. When analgesics are only administered post-operatively, there is a delay until the agent reaches an effective concentration and therefore a period of time where the animal is likely to experience pain postoperatively [14]. Administration of analgesia prior to surgery ('preemptive analgesia') may also reduce post-surgical hypersensitivity (for review see [13,14]). Although it may be inadvisable to use opioids for systemic analgesia pre- or perioperatively in combination with neuroleptanalgesic anaesthetic regimes (e.g. fentanyl and fluanisone) [13], NSAIDs or local anaesthetic agents may be used pre- or perioperatively with neuroleptanalgesic regimes. Alternatively, systemic opioids may be administered pre- or perioperatively when volatile anaesthetic regimes are used [13].

Although the majority of papers in both time periods reported some form of analgesic provision, analgesia was frequently only in the form an anaesthetic agent with analgesic properties (Table 2). This is likely to be a welfare concern as these anaesthetic agents 'may contribute to postoperative pain control but are not sufficient to exert such control in and of themselves' [14]. Another potential concern was the decreasing use in local anaesthetic agents between the two time periods (Table 2). The use of local anaesthetics such as bupivacaine by wound infiltration or regional nerve blocks, or epidural or spinal administration can provide valuable additional analgesia particularly in combination with systemic analgesic agents [13].

Multimodal analgesia is recommended in cases when pain is likely to be moderate to severe [13,14], as the administration of a single systemic analgesic agent may be insufficient to control pain resulting from procedures such as laparotomies in rabbits [15]. Unfortunately, this study does not suggest this technique is being applied to rabbits in the laboratory environment since none of the papers reported the administration of both an opioid and a NSAID.

The inclusion of animal details within the methods sections of peer-reviewed journals is important as it enables replication of studies, allows readers to judge the scientific quality of the work and because it addresses public concerns about the use of animals in research [16]. The majority of the papers included in this literature review reported the number of rabbits undergoing surgery. Studies with rabbits involved fewer animals than studies involving rats and mice; the median number of rabbits involved in a study was 22 compared to 40 in rodents [9]. The significantly higher group sizes involved in surgical studies in rodents compared to rabbits may have important welfare consequences and indicate that either: 1) the number rodents involved in surgical studies could be reduced and studies would still have sufficient statistical power or 2) the scientific quality of data with rabbits could be improved with more statistical planning. Since rodents and rabbits tend to be involved in different types of surgical studies (e.g. rabbits are frequently used in orthopaedic studies), it is difficult to form definite conclusions about whether numbers of rodents or rabbits in surgical studies may be inappropriate.

Peer-reviewed papers were less likely to report the gender of rabbits than the number of animals used in the study. In both time periods included in this review 34% of papers did not report the gender of rabbits used. Similarly 38% of chronic experimental studies published in 2000 in a range of laboratory species did not specify the gender of experimental animals used [17].

## Conclusions

Although reporting the administration of analgesic agents to rabbits undergoing potentially painful procedures increased between the two time periods examined, only half of papers published between 2005 and 2007 specified the use of a systemic analgesic agent. The literature review highlights some areas where practices in rabbit analgesia could be further improved including: routine administration of systemic analgesics, administration of systemic analgesic agents pre- or perioperatively, use of multi-modal analgesia and increased administration of non-steroidal anti-inflammatory drugs and use of other techniques such as epidural analgesia, particularly for orthopaedic procedures.

## Methods

### Search strategy

The ScienceDirect search engine http://www.sciencedirect.com was used to identify relevant studies published in English from 1995 to 1997 and from 2005 to 2007. ScienceDirect was accessed between 12-06-2008 and 30-07-2008. The search terms "skin incision"/"surgery" and "rabbit" were used and only papers that were available in electronic format at Newcastle University were selected. The materials and methods section of each paper was screened to identify whether the inclusion criteria were met.

### Inclusion criteria

The inclusion criteria were as previously described [8]. A paper was eligible for inclusion in the study if it involved the use of a rabbit in a surgical experimental procedure under general anaesthesia with a postoperative recovery procedure of at least 24 hours. Papers that: (i) did not describe methods in detail, (ii) described foetal surgery and (iii) stated efficacy of analgesia as the purpose of the study were excluded. When papers from a single group of authors described a similar series of procedures, only the first paper listed by ScienceDirect was included in the study.

Because the number of rabbits used in scientific research is decreasing, the number of the papers from 2005 to 2007 that met the inclusion criteria was the limiting factor in determining how many papers to include in the review. All of the papers from 2005 to 2007 that met the inclusion criteria were included in the review and the same search strategy was used to locate papers from 1995 to 1997. Each paper published from 1995 to 1997 was given a number based on the ScienceDirect display and a random number generator was then used to select the number of papers equivalent to the number of papers that met the inclusion criteria ten years earlier.

### Classification

Classification of procedures was as previously described [8] modified from the position statement of the American College of Laboratory Animal Medicine on recommendations for the assessment and management of pain in rodents and rabbits [18]. Each paper was classified into one of five categories: skin incision, craniotomy, laparotomy, thoracotomy or orthopaedic study. Thoracotomies and orthopaedic procedures were considered to be the most potentially painful, laparotomies were considered to be slightly less potentially painful and skin incisions and craniotomies were considered to be the least potentially painful procedures.

Anaesthetic agents were also classified according to whether they contained an analgesic component. Rabbits anaesthetised with ketamine and/or an alpha_2 _agonist (medetomidine or xylazine) were classified as having 'an anaesthetic regimen with an analgesic component' [14]. Similarly, rabbits anaesthetised with an anaesthetic combination that included a fentanyl component were also considered to have received an analgesic component in their anaesthetic [14]. With the exception of when it was used as a patch, fentanyl was not classified as a systemic analgesic because of its short-acting effect [19]

The gender of the rabbits and the total number of animals used in each study were also noted. Specification of ethical review permissions (relevant licences and/or national or institutional guidelines for the care and use of animals) were also noted.

### Statistical analyses

All statistical analyses were conducted using SPSS software (SPSS 16.0 statistical package for Macintosh, SPSS Inc., Chicago, IL, USA). A two-tailed Mann-Whitney test was used to compare the number of rabbits used in each study between time periods. Chi-squared analyses were used to compare: systemic analgesic administration, use of anaesthetic regimens with analgesic components, local anaesthetic use, use of any analgesic agent (systemic, local and/or anaesthetic with analgesic component), the severity of experimental procedures, number of papers reporting ethical approval and the gender of rabbits used between time periods and to compare whether ethical approval was reported. A value of *P *< 0.05 was considered statistically significant.

## Authors' contributions

CAC was involved in the study design, carried out the data collection and assisted with data analysis. PAF was involved in the study design and drafting the paper. MCL was involved in the study design. CAR was involved in the study design, carried out the data analysis and drafted the manuscript. All authors read and approved the final manuscript.

## Supplementary Material

Additional file 1 **Journals included in this study**. A list of journals included in this study. Click here for file
