# Supplementary material for: Reported analgesic administration to rabbits undergoing experimental surgical procedures
Source: BMC Vet Res. 2011 Feb 21;7:12. doi: 10.1186/1746-6148-7-12 (PMC3058034; doi:10.1186/1746-6148-7-12)
Supplement: Additional file 1 — Journals included in this study. A list of journals included in this study. [file 1746-6148-7-12-S1.DOC]

**Additional file 1**

**Journals included in the study**

Academic Radiology, American Heart Journal, American Journal of Obstetrics and Gynaecology, American Journal of Orthodontics and Dentofacial Orthopaedics, American Journal of Otolaryngology, Annals of Anatomy, Annals of Thoracic Surgery, Archives of Oral Biology, Asian Journal of Surgery, Atherosclerosis, Autonomic Neuroscience, Biochemica et Biophysica Acta, Biomolecular Engineering, Biomaterials, Biomedicine and Pharmacotherapy, Bone, Brain Research, British Journal of Plastic Surgery, Cardiovascular Surgery, Clinical Biomechanics, Current Applied Physics, European Journal of Cardio-thoracic Surgery, European Journal of Pharmacology, European Journal of Vascular and Endovascular Surgery, European Urology, Experimental Eye Research, Fertility and Sterility, Gastroenterology, Injury, International Immunopharmacology, International Journal of Developmental Neuroscience, International Journal of Oral and Maxillofacial Surgery, International Journal of Paediatric Otorhinolaryngology, Journal of Cranio Maxillo-Facial Surgery, Journal of Gastrointestinal Surgery, Journal of Hand Surgery, Journal of Immunological Methods, Journal of Laboratory and Clinical Medicine, Journal of Oral and Maxillofacial Surgery, Journal of Pediatric Surgery, Journal of Photochemistry and Photobiology, Journal of Shoulder and Elbow Surgery, Journal of Surgical Research, Journal of Vascular Surgery, Journal of Voice, Neuropharmacology, Neuroscience, Osteoarthritis and Cartilage, Otolaryngology, Spine Journal and Surgery.
